# RNA-Dependent RNA Polymerase of the Second Human Pegivirus Exhibits a High-Fidelity Feature

**DOI:** 10.1128/spectrum.02729-22

**Published:** 2022-08-18

**Authors:** Shuyi Chen, Jiqin Wu, Xiaofeng Yang, Qiuli Sun, Su Liu, Farooq Rashid, Emmanuel Enoch Dzakah, Haiying Wang, Jufang Wang, Peng Gong, Shixing Tang

**Affiliations:** a Department of Epidemiology, School of Public Health, Southern Medical University, Guangzhou, China; b Key Laboratory of Special Pathogens and Biosafety, Wuhan Institute of Virology, Center for Biosafety Mega-Science, Chinese Academy of Sciences, Wuhan, China; c School of Biology and Biological Engineering, South China University of Technology, Guangzhou, China; d Drug Discovery Center for Infectious Diseases, Nankai University, Tianjin, China; e Department of Molecular Biology & Biotechnology, School of Biological Sciences, College of Agriculture and Natural Sciences, University of Cape Coast, Cape Coast, Ghana; Thomas Jefferson University

**Keywords:** second human pegivirus, RNA-dependent RNA polymerase, fidelity, viral diversity

## Abstract

The virus-encoded RNA-dependent RNA polymerase (RdRp) is responsible for viral replication, and its fidelity is closely related to viral diversity, pathogenesis, virulence, and fitness. Hepatitis C virus (HCV) and the second human pegivirus (HPgV-2) belong to the family *Flaviviridae* and share some features, including similar viral genome structure. Unlike HCV, HPgV-2 preserves a highly conserved genome sequence and low intrahost variation. However, the underlying mechanism remains to be elucidated. In this study, we evaluated the fidelity of HPgV-2 and HCV RdRp in an *in vitro* RNA polymerase reaction system. The results showed higher fidelity of HPgV-2 RdRp than HCV NS5B with respect to the misincorporation rate due to their difference in recognizing nucleoside triphosphate (NTP) substrates. Furthermore, HPgV-2 RdRp showed lower sensitivity than HCV to sofosbuvir, a nucleotide inhibitor against HCV RdRp, which explained the insusceptibility of HPgV-2 to direct-acting antiviral (DAA) therapy against HCV infection. Our results indicate that HPgV-2 could be an excellent model for studying the mechanisms involved in viral polymerase fidelity as well as RNA virus diversity and evolution.

**IMPORTANCE** RNA viruses represent the most important pathogens for humans and animals and exhibit rapid evolution and high adaptive capacity, which is due to the high mutation rates for using the error-prone RNA-dependent RNA polymerase (RdRp) during replication. The fidelity of RdRp is closely associated with viral diversity, fitness, and pathogenesis. Previous studies have shown that the second human pegivirus (HPgV-2) exhibits a highly conserved genome sequence and low intrahost variation, which might be due to the fidelity of HPgV-2 RdRp. In this work, we used a series of *in vitro* RNA polymerase assays to evaluate the *in vitro* fidelity of HPgV-2 RdRp and compared it with that of HCV RdRp. The results indicated that HPgV-2 RdRp preserves significantly higher fidelity than HCV RdRp, which might contribute to the conservation of the HPgV-2 genome. The unique feature of HPgV-2 RdRp fidelity provides a new model for investigation of viral RdRp fidelity.

## INTRODUCTION

Most RNA viruses show high mutation rates, which help them escape both innate and induced host immune responses, overcome the role of antiviral therapies, and adapt to new environments through extremely rapid evolution; these abilities constitute the main obstacles to prevention and treatment of viral diseases (1). The high mutation rate of RNA viruses is mainly due to the lack of a proofreading function of viral RNA-dependent RNA polymerase (RdRp). Approximately 10^−4^ to 10^−6^ errors per nucleotide site per round of replication are produced and result in the existence of multiple viral sequences with genetic diversity (quasispecies) as well as different viral genotypes and subtypes (2–4). In contrast, a virus mutation rate within a specific error threshold could lead to the appearance of attenuated viruses or even extinction (5–8). It is believed that attenuated viruses with high-fidelity RdRp could be a potential strategy for identifying and producing viral vaccines.

The second human pegivirus (HPgV-2) is a single-stranded positive-sense RNA virus and belongs to the genus *Pegivirus* of the family *Flaviviridae*. It was discovered from the plasma of hepatitis C virus (HCV)-infected patients in 2015 and proved to be epidemiologically associated with and structurally similar to HCV (9–11). In contrast to HCV and the first human pegivirus (HPgV-1, or GBV-C), HPgV-2 exhibits minimal genetic diversity, and identity of HPgV-2 genetic sequences collected from different countries or regions is as high as 94%, while both HCV and HPgV-1 have multiple genotypes and subtypes (12). In addition, HPgV-2 shows a significantly lower level of quasispecies and intrahost variation than HCV (13). The high conservation of the HPgV-2 genome may be related to inefficient innate immune responses and low immune selection pressures (12, 13). Previous studies suggest that HPgV-2 may be an ancient and stable virus with many fewer mutations. Furthermore, HPgV-2 is well adapted to the host environment and can efficiently escape the immune response. Therefore, HPgV-2 provides us with a new model to investigate the mechanism of the evolution and genetic diversity of RNA viruses. However, the contribution of HPgV-2 RdRp to its genome conservation remains poorly studied.

Moreover, our previous studies indicated that direct-acting antiviral (DAA) treatment, such as the nucleotide inhibitor (NI) sofosbuvir targeting viral RdRp, could eliminate HCV but not HPgV-2 infection (13, 14). The preclinical assessments and clinical analysis of sofosbuvir trials have shown that mutations in HCV RdRp lead to a decrease in drug susceptibility (15–18) and that the drug resistance is closely related to RdRp fidelity (19, 20). The different susceptibilities of HCV and HPgV-2 to sofosbuvir provide us with a possibility for exploring the mechanism of the interaction between sofosbuvir and viral RdRp and a model to analyze potential variants with resistance to sofosbuvir or other compounds targeting viral RdRp.

Therefore, as RdRp is the critical enzyme for virus replication, fidelity of viral RdRps is important not only for studies on viral diversity, fitness, and evolution but also for development of antiviral therapies and vaccines. In this study, we explored and compared RdRp fidelity of HPgV-2 and HCV by establishing *in vitro* RdRp assays and revealed higher RdRp fidelity of HPgV-2 than HCV, both in regular nucleotide incorporation and in the usage of the nucleoside triphosphate (NTP) form of sofosbuvir. The unique features of HPgV-2 RdRp make it an excellent model for investigating RNA virus diversity and drug-resistant variants and designing RdRp fidelity-attenuated viral vaccines.

## RESULTS

### Establishment of *an in vitro* RdRp assay for HPgV-2 NS5B.

**FIG 1:**
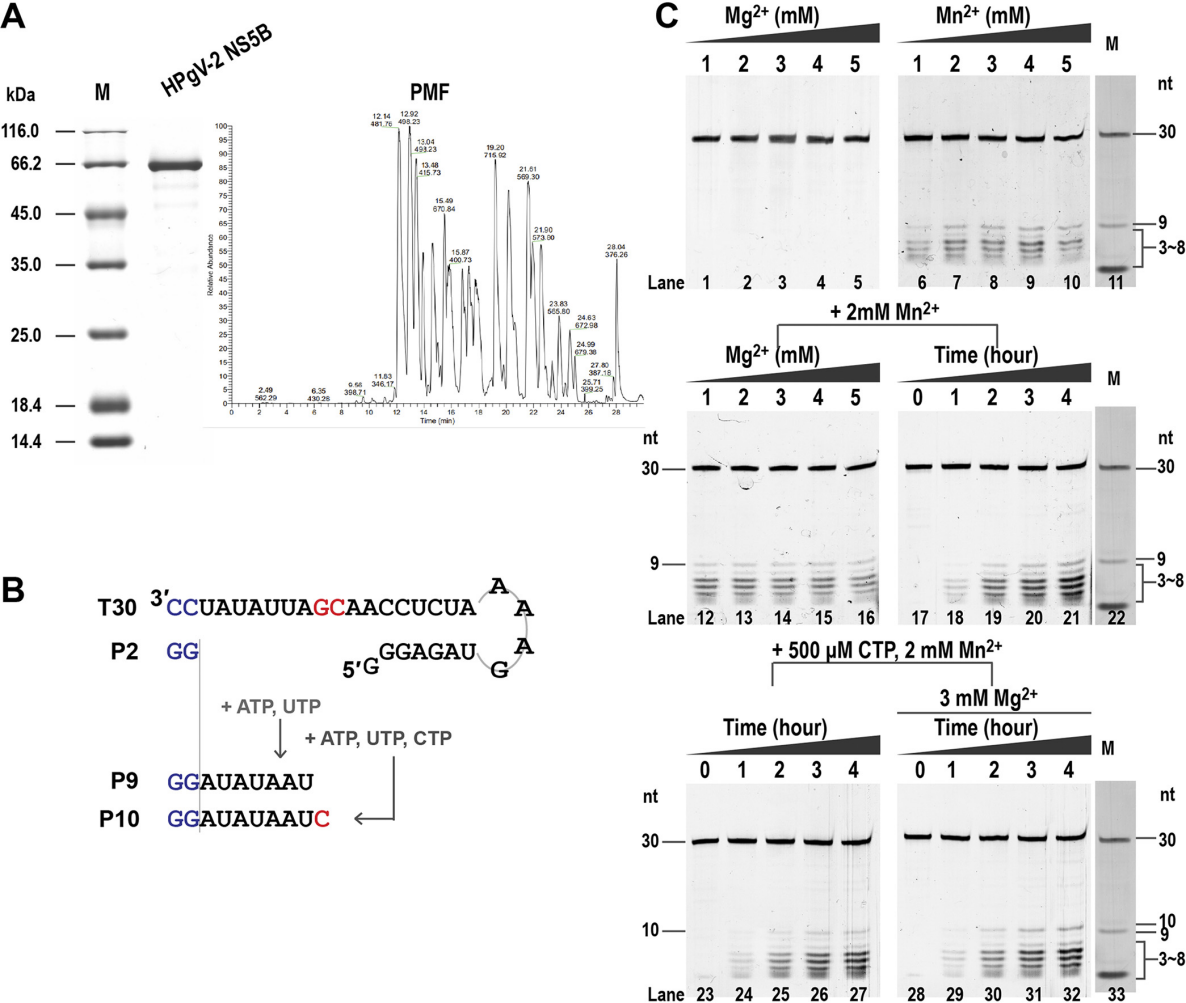
Characterization of *in vitro* polymerase activity of HPgV-2 NS5B. (A) SDS-PAGE and PMF analysis of the purified HPgV-2 NS5B. (B) Schematic diagram of the *in vitro* RNA polymerase assay. The RNA template T30 was annealed with the dinucleotide primer P2 before the reaction. When ATP and UTP were supplied as the only NTP substrates, the P2 primer directed the extension of a 7-nucleotide fragment to produce a 9-mer product (P9) following the principle of complementary base pairing and using T30 as the template. When ATP, UTP, and CTP were used as substrates, a 10-mer product (P10) was synthesized. (C) Reaction conditions of divalent cation and incubation time for the HPgV-2 NS5B *in vitro* polymerase assay. For lanes 1 to 10, the reactions were performed in the presence of Mg^2+^ or Mn^2+^ at the indicated concentrations for a 2-h incubation. For lanes 12 to 16, the reactions were performed in the presence of 2 mM Mn^2+^ and Mg^2+^ at the indicated concentrations for a 2-h incubation. The products catalyzed by HCV NS5B were used as markers, in which there are obvious bands of the 9-nt and 3-nt products.

After purification and authentication by peptide mass fingerprinting (PMF) (Fig. 1A), HPgV-2 RdRp NS5B was subjected to the *in vitro* polymerase reaction to confirm its catalytic activity and to determine the optimum reaction conditions. Fig. 1B shows the reaction scheme used to test the activity of HPgV-2 NS5B, in which the preannealed T30/pGG construct and the NS5B were mixed with ATP/UTP or ATP/UTP/CTP as substrates in an optimized reaction buffer to start the RNA synthesis. Directed by the sequence of 30-nucleotide (nt) template RNA T30, HPgV-2 RdRp was expected to produce 9-mer (P9) or 10-mer (P10) products with such NTP combinations. While magnesium ion (Mg^2+^) was sufficient as the only divalent metal ion for *de novo* polymerase activity of HCV NS5B, we found that manganese (Mn^2+^), but not Mg^2+^, was essential for HPgV-2 NS5B activity and ≥2 mM Mn^2+^ could significantly increase P9 production (Fig. 1C, lanes 1 to 10). In the presence of 2 mM Mn^2+^, the concentration of Mg^2+^ did not affect the production of P9 (Fig. 1C, lanes 12 to 16). We also observed 3- to 8-mer intermediate products in the HPgV-2 NS5B polymerase reaction, while the amount of the 3- to 8-mer and P9 products increased when the reaction time was extended to 3 and 4 h at 30°C (Fig. 1C, lanes 17 to 21). These results suggest that the 3- to 8-mer products might be the abortive reaction products that were prematurely released from the enzymes. Furthermore, as expected, HPgV-2 NS5B catalyzed the production of P10 when ATP, UTP, and CTP were used as substrates and incubated at 30°C for more than 2 h with or without Mg^2+^ (Fig. 1C, lanes 23 to 32). Therefore, our results confirmed the catalytic ability of HPgV-2 NS5B to synthesize the expected products in the presence of Mn^2+^. The reaction condition with Mn^2+^ was thus used in the subsequent investigation to assess the fidelity of HPgV-2 NS5B.

### Higher fidelity of HPgV-2 NS5B than HCV NS5B in the *in vitro* RdRp assay.

**FIG 2:**
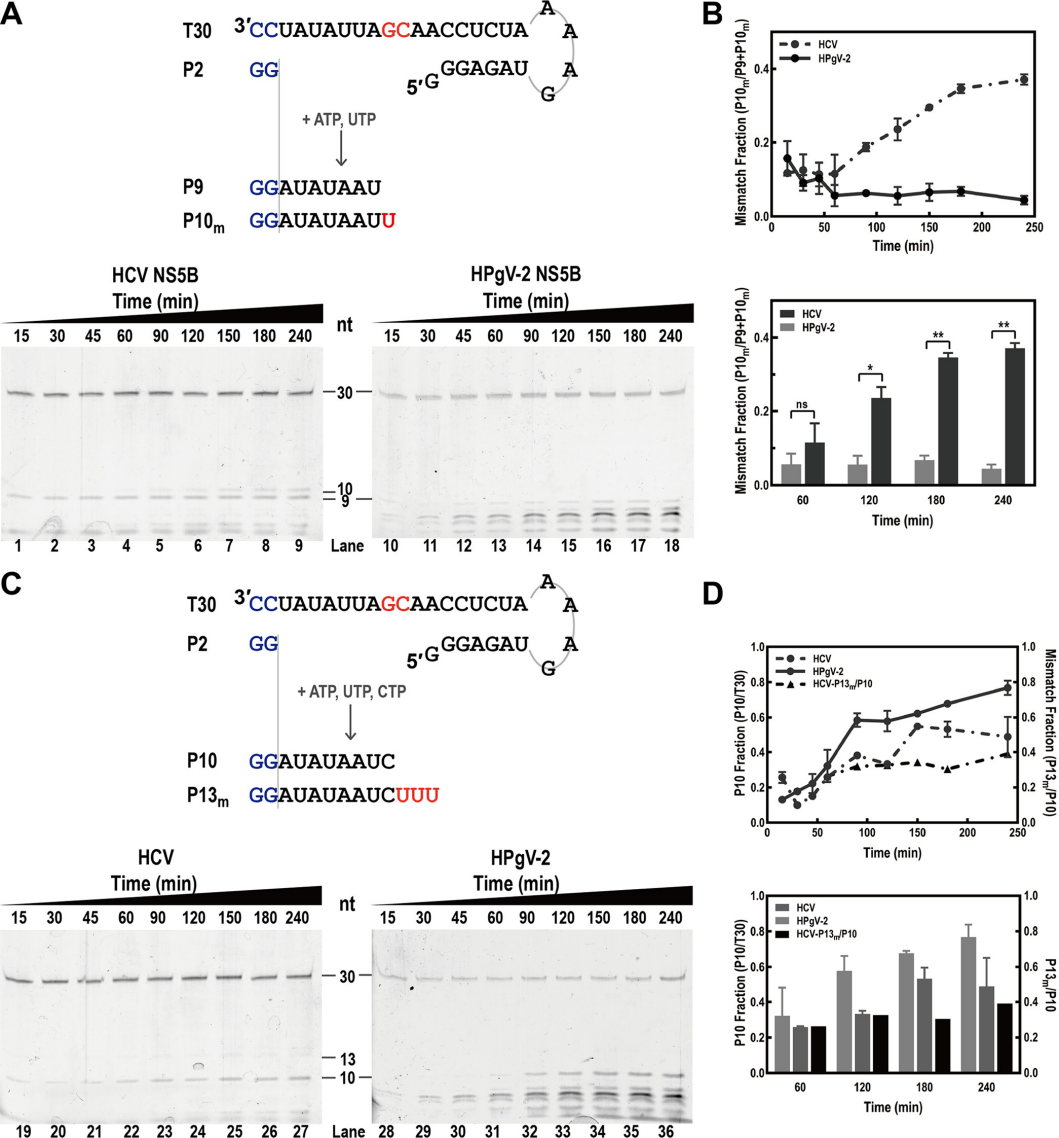
Comparison of the fidelity of HPgV-2 NS5B and HCV NS5B using a one-step polymerase catalytic assay. (A) The misincorporation product P10_m_ was produced in a pattern of G:U mismatch when ATP and UTP were the only NTP substrates. (B) The intensity ratio P10_m_/(P9+P10_m_) was plotted in a line chart to reflect the increasing trend of P10_m_ when the incubation time was extended. The values of P10_m_/(P9+P10_m_) at 60, 120, 180, and 240 min postreaction are shown in the column chart. *, *P* < 0.05; **, *P* < 0.005; ns, no significant difference (*t* test). (C) Synthesis efficiency of P10 when ATP, UTP, and CTP were provided as substrates. The correct product, P10, and the mismatched product, P13_m_, were monitored at different time points. (D) The synthesis efficiency and misincorporation level are presented as the intensity ratios P10/T30 and P13_m_/P10, respectively, and plotted in a line chart or shown in the column chart. The *t* test analysis showed that there was no significant difference between HPgV-2 and HCV NS5B in the P10 fraction.

We conducted an *in vitro* polymerase reaction to elucidate and compare the fidelity of HPgV-2 NS5B with HCV NS5B. The ratio of P10_m_/(P9+P10_m_) (P10_m_ is a misincorporation-derived P10 product) was used for preliminary evaluation of the fidelity of viral RdRp, since the misincorporation caused by the error-prone viral RdRp resulted in the production of P10_m_ in addition to the expected P9 product in the presence of the T30/pGG construct (Fig. 2A). The RNA synthesis was monitored at the indicated incubation time points. HPgV-2 NS5B showed much less misincorporation, as the P10_m_/(P9+P10_m_) ratio was less than 0.1 for HPgV-2 NS5B and ~0.4 for HCV NS5B even at 240 min, while the P10_m_/(P9+P10_m_) ratio significantly increased for HCV NS5B but not for HPgV-2 NS5B as the reaction proceeded (Fig. 2A and B). Further, we measured the ratio of P10/T30 by calculating the amount of the correct product P10 in the presence of the substrates ATP, UTP, and CTP (Fig. 2C and D). HPgV-2 NS5B was able to produce P10 at incubation times longer than 30 min, and the amount of P10 obviously increased dependent on reaction time. Almost no P13_m_ misincorporation product was observed for HPgV-2 NS5B, while the level of P13_m_ gradually increased in the presence of HCV NS5B after 60 min (Fig. 2C and D). Taken together, these results indicate that HPgV-2 NS5B is less prone to misincorporation of the substrates than HCV NS5B.

### Higher fidelity of HPgV-2 NS5B than HCV NS5B in an *in vitro* single-nucleotide-incorporation assay.

**FIG 3:**
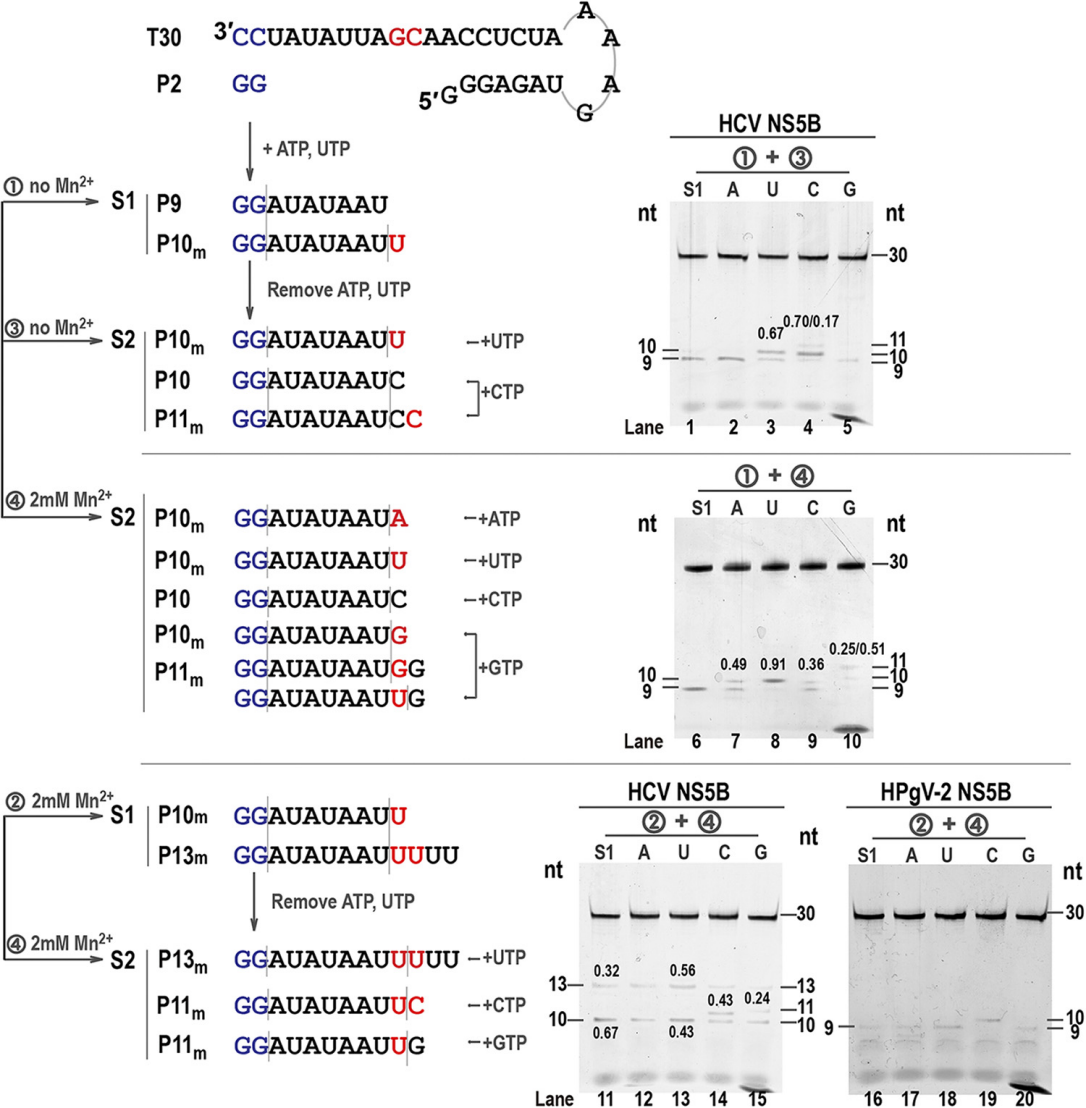
Comparison of the fidelity of HPgV-2 NS5B and HCV NS5B in the single-nucleotide-incorporation assay. The assay was performed in a two-step pattern in which P9 was synthesized in step 1 and subsequently extended to P10, P11, and P13 in step 2 in the presence of different NTP substrates. The fidelity of HCV NS5B was assessed in the absence of Mn^2+^ in steps 1 and 2 (conditions 1 and 3 [circled numbers]), in the absence of Mn^2+^ in step 1 and in the presence of Mn^2+^ in step 2 (conditions 1 and 4), or in the presence of Mn^2+^ in both steps 1 and 2 (conditions 2 and 4). The fidelity of HPgV-2 NS5B was assessed in the presence of Mn^2+^ in both steps 1 and 2 (conditions 2 and 4) by calculating the ratio of the corresponding misincorporated products to the total RNA products.

Since the levels of misincorporation could be affected by the sequence of the mismatched sites and the type of misincorporation in the polymerase reaction, we adapted a single-nucleotide-incorporation assay to get further insight into the fidelity of HPgV-2 and HCV NS5B. The misincorporation was assessed in a two-step single-nucleotide-incorporation assay (Fig. 3). In step 1, only ATP and UTP were provided as substrates, the reaction was paused after the synthesis of the P9 product, and the NS5B-T30-P9 complexes were assembled due to the absence of CTP. The above-described reaction mixture was then centrifuged to pellet the NS5B-T30-P9 complexes and to remove the original ATP and UTP substrates. In step 2, one type of NTP was provided as the only substrate in each reaction to monitor the conversion of the P9 product to longer products and to individually assess the incorporation of each of the four NTPs. Since Mn^2+^ was essential for HPgV-2 NS5B activity, we also investigated the effect of Mn^2+^ on the misincorporation level caused by HCV NS5B in this assay.

Our results showed that higher misincorporation level occurred in G:U_mis_ pattern and was caused by HCV NS5B either in the presence or absence of Mn^2+^ in steps 1 and 2. For example, the ratio of the mismatched fraction of UTP for HCV NS5B over the total products was 0.67 in the absence of Mn^2+^ (Fig. 3, conditions 1 and 3 [circled numbers], lane 3) and increased to 0.91 when 2 mM Mn^2+^ was added in step 2 (Fig. 3, conditions 1 and 4, lane 8) in a 2-h reaction. Supplementation of 2 mM Mn^2+^ in step 1 resulted in significant increase of the mismatched product P13_m_, and the G:U mismatch reached a ratio of 0.56 (Fig. 3, conditions 2 and 4, lane 13). In addition, single CTP also resulted in misincorporation and produced the C:C_mis_ fraction P11_m_ for HCV NS5B. The ratio of P11_m_ to the total products was 0.17 in the absence of Mn^2+^ (Fig. 3, conditions 1 and 3, lane 4) and 0.36 in the presence of Mn^2+^ (Fig. 3, conditions 1 and 4, lane 9). When Mn^2+^ is present in both steps 1 and 2, the majority of P10_m_ and P13_m_ products were generated even in step 1, and P10_m_ was mainly converted into the C:C_mis_-derived P11_m_ and the C:U_mis_-derived P13_m_ in step 2 but hardly showed the C:A_mis_-derived product profile for HCV NS5B (Fig. 3, conditions 2 and 4, lanes 11 to 15). Thus, according to the susceptibility of HCV NS5B to the misincorporation of a single NTP, the crude ranking order for NTPs was UTP > CTP > GTP > ATP, considering the differences in template nucleotide and/or nucleotide combination. In sharp contrast, even in the presence of 2 mM Mn^2+^ in steps 1 and 2 and four types of NTPs in step 2, no significant mismatched products were observed for HPgV-2 NS5B (Fig. 3, conditions 2 and 4, lanes 16 to 20). These results were consistent with the findings of the above-described polymerase assay, indicating that HPgV-2 NS5B possesses higher fidelity than HCV NS5B. As the presence of Mn^2+^ would obviously increase the misincorporation for HCV NS5B, in the following two-step assay, reactions for HCV NS5B were performed in the absence of Mn^2+^ (condition 1 and 3). Also, G:U_mis_ would be used in the following single-nucleotide-misincorporation assay for simplicity because of the highest misincorporation susceptibility for HCV NS5B.

**FIG 4:**
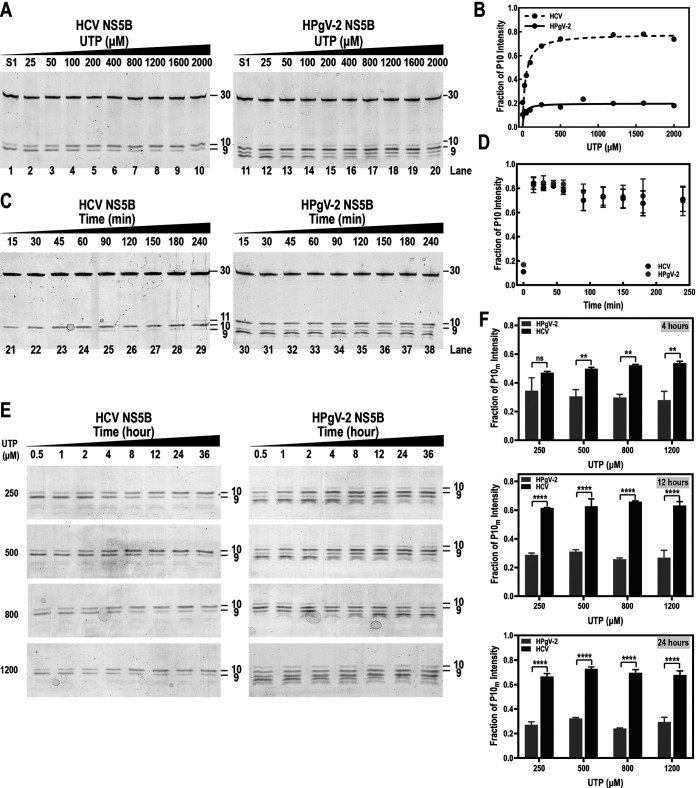
Evaluation of the fidelity of HPgV-2 NS5B over substrate concentrations and reaction times. (A) UTP at a series of concentrations (25 μM to 2,000 μM) were supplied in the step 2 reaction of the G:U_mis_ misincorporation assay to assess the misincorporation using 20% urea-PAGE for HCV and HPgV-2 NS5B. (B) The fraction of P10_m_ intensity (*y* axis) was quantified and plotted against UTP concentration (*x* axis) for HCV (dashed line) or HPgV-2 NS5B (solid line). (C) CTP (500 μM) was supplied as the only substrate in the step 2 reaction in a time course manner. The products were detected with 20% urea-PAGE for HCV and HPgV-2 NS5B. (D) The intensity of P10 in panel C was quantified at each time point (*y* axis) and plotted against the reaction time (*x* axis) for HCV (black dots) or HPgV-2 NS5B (gray dots). (E) Different concentrations of UTP (250, 500, 800, and 1,200 μM) were added as the only substrate in the step 2 reaction, and the reaction was performed in a time course manner (incubated for 0.5, 1, 2, 4, 8, 12, 24, and 36 h). The misincorporated product P10_m_ was analyzed using 20% urea-PAGE electrophoresis. (F) The intensity of P10_m_ at 4, 12, and 24 h of incubation with different UTP concentrations was quantified. *, *P* < 0.05; **, *P* < 0.005; ****, *P* < 0.0001; ns, no significant difference (*t* test).

Next, we investigated whether the concentration of UTP and reaction time would affect the level of G:U_mis_ misincorporation. When different concentrations of UTP were added in the step 2 reaction (Fig. 4A), we found that the misincorporated P10_m_ product catalyzed by HCV NS5B gradually accumulated and the ratio of P10_m_ to the total P9+P10_m_ products reached approximately 0.8 in the presence of 400 μM UTP (Fig. 4A and B). However, the amount of P10_m_ produced by HPgV-2 NS5B was maintained at relatively stable levels of 0.1 to 0.2 regardless of the concentration of UTP (Fig. 4A and B), suggesting that increasing the NTP substrate concentration would not aggravate the fidelity of HPgV-2 NS5B. In order to further ascertain the fidelity of HPgV-2 NS5B, the single-nucleotide G:U_mis_ misincorporation analysis utilizing a series of concentrations of UTP in a time course manner was performed. As results, the portion of the incorporation product P10_m_ of HPgV-2 NS5B was maintained at a very low level, no higher than 0.4, independent of the UTP concentration and incubation time, while those of HCV NS5B were increased from approximately 0.3 to 0.8 upon increase of UTP concentration and time (Fig. 4E and F). As a control, a time course reaction was performed with CTP as the only substrate at 500 μM. As expected, there was no significant difference in the synthesis of the correct P10 between HCV and HPgV-2 NS5B (Fig. 4C and D). At this point, we affirmed that HPgV-2 NS5B may possess rarely high fidelity among viral RdRps, which is relatively independent of the substrate concentration and reaction time.

### Lower sensitivity of HPgV-2 NS5B to sofosbuvir than HCV NS5B.

**FIG 5:**
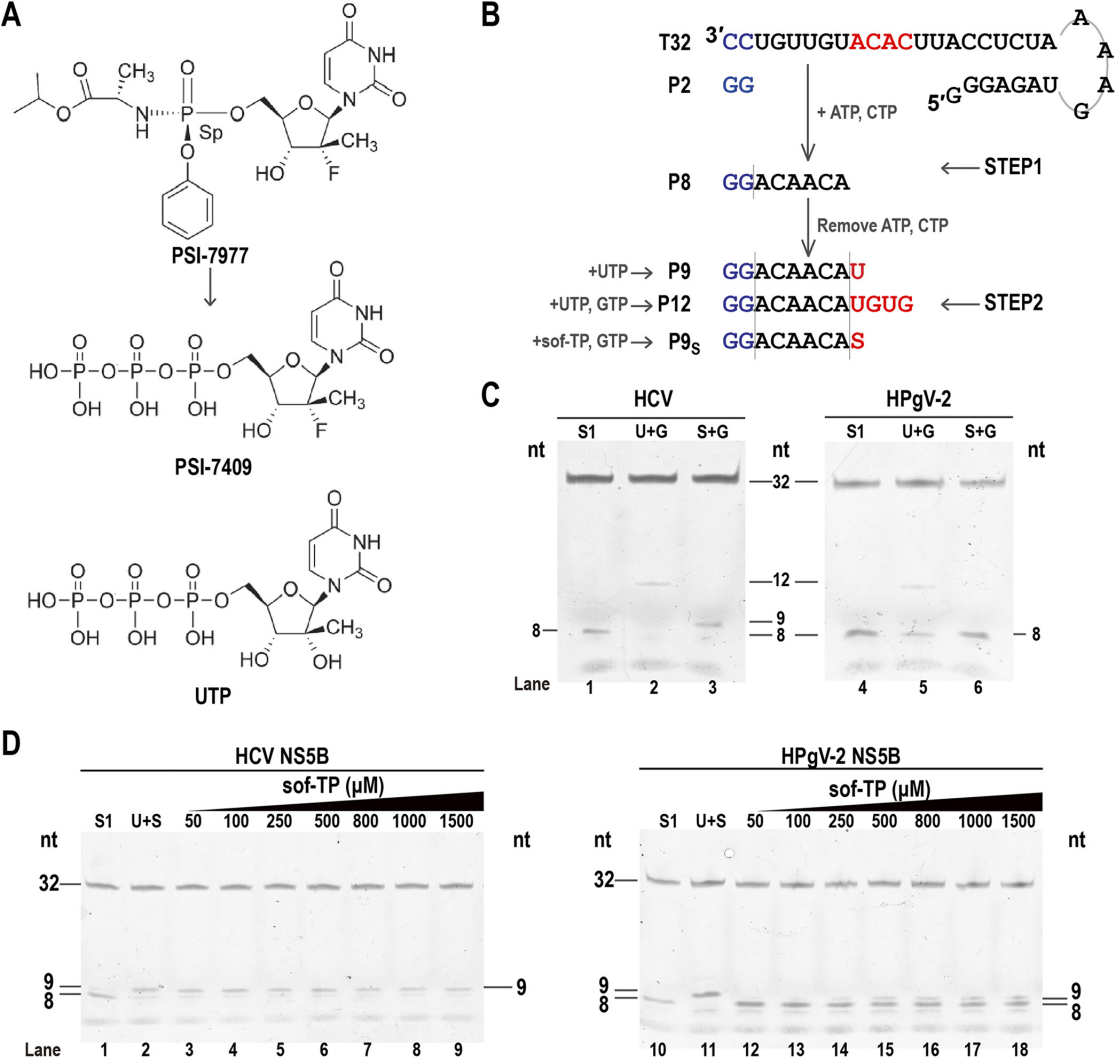
Interaction of sofosbuvir with NS5B of HPgV-2 and HCV. (A) Structures of monophosphate sofosbuvir (PSI-7977), triphosphate sofosbuvir (PSI-7409; sof-TP), and UTP. (B) Schematic diagram of the two-step single-nucleotide-incorporation assay. (C) Urea-PAGE for RNA products synthesized by HCV NS5B (left) and HPgV-2 NS5B (right) in step 1 (S1) and step 2 in the presence of UTP and GTP (U+G) or sof-TP and GTP (S+G). The numbers 8, 9, 12, and 32 represent RNA products P8, P9s, and P12 as well as RNA template T32, respectively. (D) Urea-PAGE for the dose-response reaction of sofosbuvir. RNA products synthesized by HCV NS5B (left) and HPgV-2 NS5B (right) in step 1 (S1) and in step 2 in the presence of UTP and sof-TP (U+S) or different concentrations of sof-TP.

Our previous studies showed that sofosbuvir can inhibit HCV replication but not HPgV-2 replication (13, 14). Sofosbuvir is a uridine analog and a nucleotide inhibitor (NI) of RdRp for the treatment of chronic HCV infection (21–23). Once the monophosphate derivative of sofosbuvir (PSI-7977) is phosphorylated to become the active triphosphate form of sofosbuvir (PSI-7409; sof-TP) (Fig. 5A), it competes with natural UTP to terminate RNA elongation through steric hindrance exerted by 2′-fluoro groups (24) and to inhibit virus replication by targeting the highly conserved active site of RNA polymerase (23, 25). Here, we carried out a comparative analysis of sofosbuvir selectivity in HPgV-2 and HCV RdRps.

We adapted a two-step single-nucleotide-incorporation assay by using T32 as the RNA template and pGG as the primer (Fig. 5B). Both HCV and HPgV-2 NS5B successfully synthesized an 8-mer product (P8) in step 1 and a 12-mer product (P12) in step 2 in the presence of UTP+GTP (Fig. 5C, lanes 1, 2, 4, and 5). When sof-TP and UTP were supplied, P9_S_ (“s” denotes the misincorporated sofosbuvir) product was found only in the system with HCV NS5B and not that with HPgV-2 NS5B (Fig. 5C, lanes 3 and 6), indicating that sof-TP was not able to incorporate by HPgV-2 NS5B. The dose-response reaction results indicated that a clear P9_S_ band was observed in the presence of HCV NS5B at a low sof-TP concentration of 50 μM, while a very weak P9_S_ band existed in the presence of HPgV-2 NS5B even at 250 μM sof-TP (Fig. 5D, lanes 3 and 14). Although a relatively large amount of P9_S_ was observed in the presence of HPgV-2 NS5B at 500 μM sof-TP, the amount of P9_S_ did not increase further in the presence of higher concentrations (800 μM, 1,000 μM, and 1,500 μM) of sof-TP (Fig. 5D, lanes 15 to 18). These results indicated lower affinity of HPgV-2 NS5B for sofosbuvir than HCV NS5B.

## DISCUSSION

In this study, we confirmed the significantly higher fidelity of viral RdRp of HPgV-2 than that of HCV in *in vitro* RdRp assays. We further proved that HPgV-2 RdRp could not efficiently catalyze the incorporation of the NI drug sofosbuvir even at an extremely high concentration (Fig. 5D, lane 18, 1,500 μM), possibly due to differences in the sof-TP interaction with the RdRps of HPgV-2 and HCV. Our results confirm the unique feature of HPgV-2 with respect to its high fidelity of RdRp, which likely contributes to low viral genetic diversity.

The high mutation frequency of RNA viruses is thought to be essential for rapid evolution and adaption. The molecular basis of the high mutation frequency relies on the error-prone RdRp, which produces 10^−4^ to 10^−6^ errors per nucleotide site (4, 26). Increasing viral RdRp fidelity may restrain not only viral genetic diversity but also virulence and fitness. Variants with high-fidelity RdRp show basal mutation rates approximately 5- and 2-fold lower than that of their wild-type counterparts, and most of them have decreased fitness and attenuated virulence *in vivo*, suggesting that high fidelity of viral RdRp results in a less diverse RNA virus population, which in turn is unable to produce potentially advantageous adaptive mutations (7, 27–30). Our study indicated that HPgV-2 NS5B produced significantly fewer misincorporated products than HCV NS5B in *in vitro* polymerase assays, suggesting the high fidelity of HPgV-2 RdRp (Fig. 2 to 4). The high fidelity of HPgV-2 RdRp may explain less viral diversity and high conservation of HPgV-2 genome sequences worldwide (9–12, 31). Although the possible consequence of increasing RdRp fidelity is viral attenuation and inefficient adaptation, as mentioned above, the association between the increased RdRp fidelity and the lack of pathogenesis and low prevalence of HPgV-2 remains to be elucidated. HPgV-2 is very rare and is infrequently detected in healthy blood donors, but it is closely associated with active HCV infection. Forberg et al. suggested that the relationship between HPgV-2 and HCV is commensal but not dependent (12). It is hypothesized that, with reliance on HCV, HPgV-2 would be under minimal selective pressure and maintain its sequence fidelity within the host (13). We emphasize that as the fidelity of HPgV-2 RdRp was evaluated only in *in vitro* polymerase assays, our results may not completely reflect the true behavior of HPgV-2 RdRp *in vivo.* Given the unique feature of HPgV-2 RdRp, HPgV-2 may be a specific model for investigating RNA virus diversity, replication, and evolution.

**FIG 6:**
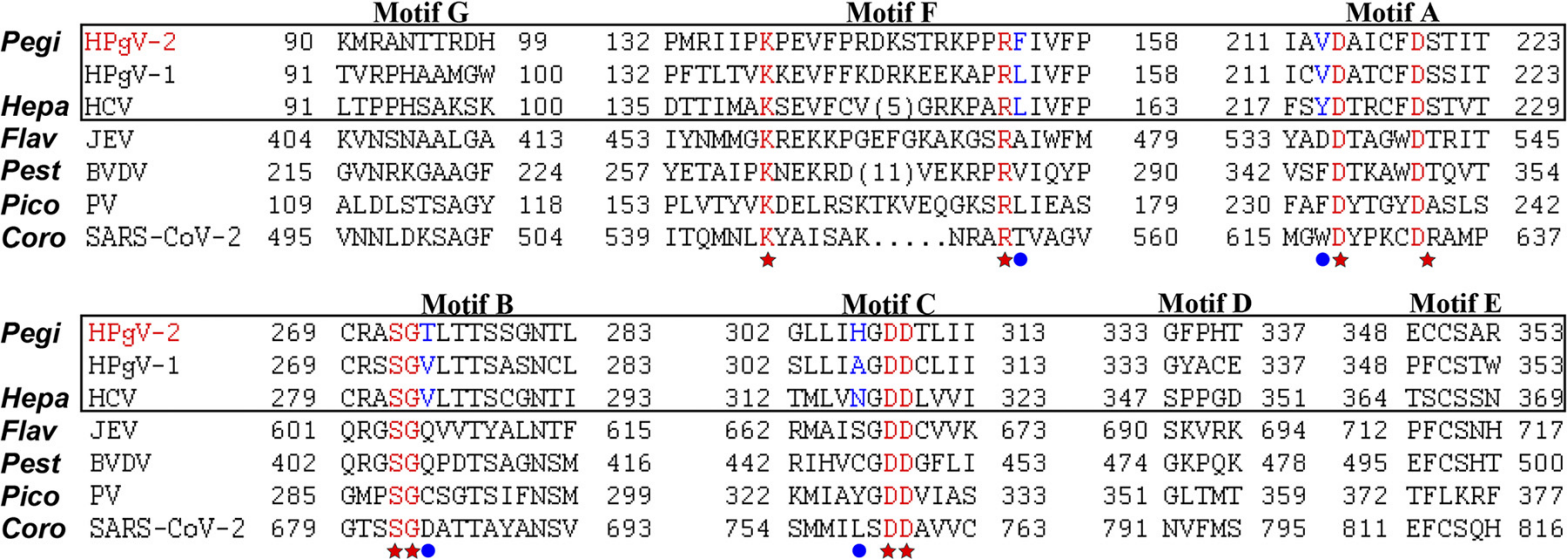
Candidate HPgV-2 RdRp residues proposed to be involved in fidelity regulation. Structure-based alignment of motifs A to G of RdRps from pegiviruses (Pegi) HPgV-2 and HPgV-1, the hepacivirus (Hepa) HCV (PDB code 1NB4), the flavivirus (Flav) Japanese encephalitis virus (JEV; PDB entry 4K6M), the pestivirus (Pest) bovine viral diarrhea virus (BVDV; PDB entry 1S4F), the picornavirus (Pico) poliovirus (PV; PDB entry 3OL6), and the coronavirus (Coro) severe acute respiratory syndrome coronavirus 2 (SARS-CoV-2; PDB entry 7C2K). Highly conserved residues are in red and indicated by stars. Residues suggested as fidelity-regulating candidates in HPgV-2 RdRp are indicated by circles.

RdRps share seven structurally conserved motifs, designated A to G, which play key roles in genome replication and fidelity control. Structure and biochemistry data from PV and HCV catalytic complexes have shown that RdRps adopt a unique palm-based active site closure to achieve phosphodiester bond formation (19, 32, 33). Highly conserved residues exist in motifs A, B, C, and F (Fig. 6, stars) to accommodate the NTP binding and to facilitate active site closure. Sequence alignment analysis and biochemistry data indicate that these motifs likely contain variant residue adjacent to their highly conserved neighboring residues to regulate RdRp fidelity (Fig. 6, circles) (34–37). Compared to HCV RdRp motif sequences, residues F154 in motif F (adjacent to invariant R153), V213 in motif A (adjacent to invariant D214), T273 in motif B (adjacent to highly conserved SG sequence), and H306 in motif C (adjacent to highly conserved XDD sequence) are candidates of interest. Comparative investigation of these sites in HPgV-2 and HCV RdRps is necessary to test their relevance to fidelity regulation.

Nucleotide inhibitors, like ribavirin, sofosbuvir, and remdesivir, are widely used as antiviral agents targeting viral RdRps. Resistance to these drugs often yields virus variants harboring mutations in the RdRp that affect replication fidelity. In the preclinical and clinical assessments of sofosbuvir trials, drug-resistant HCV variants showed mutations in the viral RdRp, such as S282T, L159F, and V321A (17, 18). In our studies, we found that HPgV-2 is insensitive to sofosbuvir in HPgV-2-infected patients and HPgV-2 RdRp is resistant to the triphosphate form of sofosbuvir in the *in vitro* polymerase reaction (Fig. 5), indicating the unique features of HPgV-2 RdRp. These findings will deepen our understanding of the relationship between RdRp fidelity and drug resistance. As mentioned above, alterations of viral RdRp fidelity always result in virulence attenuation and inefficient adaptation. The variants with high fidelity of RdRp may serve as potential attenuated vaccine strains (38). However, the amino acid sequences and structures that affect RdRp fidelity greatly differ in RNA viruses and should be further analyzed.

The mechanisms that regulate fidelity of RdRp will be useful to guide the rational design of high/low-fidelity RdRp mutants and the development of attenuated vaccines. There are approximately 1.5 to 2 million new HCV infections worldwide each year. The World Health Organization (WHO) has set a goal of eliminating HCV by 2030, and a preventive HCV vaccine will be critical to the achievement of this target. However, a HCV vaccine is still unavailable. HPgV-2 is not only structurally similar to but also epidemiologically linked with HCV. The unique feature of the high fidelity of HPgV-2 RdRp may give us new ideas for developing an HCV live attenuated virus vaccine. We believe that further investigation of HPgV-2 RdRp has great practical significance for elucidating RNA virus diversity and evolution, as well as for design of attenuated virus strains.

## MATERIALS AND METHODS

### Nucleic acids.

All NTPs and dNTPs were of ultrapure grade and purchased from the New England BioLabs, Inc. (Ipswich, USA). The RNA templates T30 and T32 and the 5′-phosphorylated dinucleotide primer pGG were synthesized and purified to high-performance liquid chromatography (HPLC) grade by TaKaRa Biomedical Technology (Kusatsu, Japan). The triphosphate metabolite of sofosbuvir PSI-7409 was purchased from MedChemExpress (New Jersey, USA) with a purity of ≥95.0%.

### Plasmid construction and protein expression.

DNA corresponding to the HPgV-2 NS5B residues 1 to 551 (QSYN to GASR) and HCV NS5B residues 1 to 570 (SMSY to ARFR) were amplified from HPgV-2 cDNA (GenBank accession number AUW64509.1) and HCV cDNA (GenBank accession number KT735184.1) and cloned into the pET-26b vector. The recombinant plasmid was transformed into Escherichia coli BL21(DE3) for overexpression. The transformed E. coli colonies were transferred to LB broth medium containing 50 μg/mL kanamycin and incubated overnight at 37°C with shaking at 250 rpm. The overnight culture was diluted 1:100 in LB broth medium containing kanamycin and grown to an optical density at 600 nm (OD_600_) around 0.5 before the addition of isopropyl-β-d-thiogalactoside (IPTG) to a final concentration of 0.2 mM, and the cells were grown overnight at 16°C to induce protein expression.

### Protein purification.

The protein was expressed with a C-terminal His_6_ tag. The purification of His tag proteins was performed by Ni^2+^ affinity chromatography. Briefly, the recombinant E. coli pellet was suspended in binding buffer (50 mM Tris-Cl, 500 mM NaCl, 30 mM imidazole [pH 8.0]). Cells were disrupted by passing through a D-3L high-pressure homogenizer (PhD Technology International, Minnesota, USA) at 15,000 lb/in^2^, and the lysate was centrifuged at 16,700 × *g* for 30 min at 4°C. The supernatant was applied to a nickel-charged HisTrap HP column (5 mL; GE Healthcare, Pittsburgh, PA, USA), and the bound protein was eluted using elution buffer (50 mM Tris-Cl, 500 mM NaCl, 500 mM imidazole [pH 8.0]) in a linear gradient from 0 mM to 500 mM imidazole for 20 column volumes (CVs). The fraction containing the target protein was desalted in starting buffer (25 mM MES [morpholineethanesulfonic acid], 0.5 mM EDTA [pH 6.0]) with a HiPrep 26/10 desalting column (GE Healthcare, Pittsburgh, PA, USA) and subsequently loaded onto a HiTrap SP HP column (5 mL; GE Healthcare, Pittsburgh, PA, USA), followed by eluting with SP elution buffer (25 mM MES, 1 M NaCl, 0.5 mM EDTA [pH 6.0]) in a linear gradient to from 0 M to 1 M NaCl for 20 CVs. The protein was then concentrated and further purified by size exclusion chromatography using a Sephacryl S-200 high-resolution column (750 mL; GE Healthcare, Pittsburgh, PA, USA) equilibrated with storage buffer (50 mM Tris-Cl, 20 mM NaCl, 20% glycerol [pH 7.0]). The purified protein was confirmed by sodium dodecyl sulfate-polyacrylamide gel electrophoresis (SDS-PAGE) analysis and PMF. Both HPgV-2 and HCV NS5B were concentrated to approximately 10 mg/mL and then aliquoted and stored at −80°C.

### *In vitro* polymerase assay.

The RNA template T30 was annealed with pGG primer at a 1:10 molar ratio at 95°C for 5 min and slowly cooled at room temperature to form the T30/pGG dimer. The *in vitro* polymerase reaction was conducted in an optimized reaction buffer (25 mM MES [pH 6.5], 20 mM NaCl, 5 mM dithiothreitol [DTT], 3 mM MgCl_2_/2 mM MnCl_2_) containing 3 μM NS5B, 2 μM T30/pGG, and 500 μM ATP and UTP and incubated at a 30°C water bath for 2 h. The concentration of divalent metal ions and the reaction time courses are given in the figure legends when necessary. To stop the reaction, 2× stop solution (95% formamide, 5 mM EDTA, 0.1% bromophenol blue, 0.1% xylene cyanol) was mixed with the samples and heat denatured for 5 min at 95°C. Then the samples were subjected to 20% urea denaturing polyacrylamide gel electrophoresis (urea-PAGE), and the gels were stained with Stain-All (Sigma-Aldrich, USA) for detection and quantification of RNA products.

### Single-nucleotide-incorporation assay.

The single-nucleotide-incorporation assay was performed in a two-step reaction. In the first step, the reaction was carried out as described above by using 500 μM ATP and UTP as substrates. After 2 h of incubation at 30°C, the reaction mixtures were centrifuged at 17,000 × *g* for 5 min, and the supernatant was discarded. The pellet was washed twice with 25 mM MES buffer (pH 6.5). In the second step, the elongation complex was resuspended in the reaction buffer with 500 μM NTP. Subsequently, the samples were incubated at 30°C for another 2 h. For the UTP misincorporation experiments, the concentration of UTP and the reaction time course are indicated in the figure legends. To investigate the role of sofosbuvir, T32 was used as an RNA template and 500 μM ATP and GTP were used as substrates in the step 1 reaction, while UTP or sofosbuvir was added as a substrate in the step 2 reaction. After reactions, all the samples were subjected to 20% urea-PAGE, and the RNA was visualized by Stain-All staining.

### RNA product quantification.

The intensity of the corresponding RNA products was measured by ImageJ software. In the one-step polymerase assay with ATP and UTP as substrates, the intensity of the mismatch fraction P10_m_ was estimated as P10_m int_/P9_int_ + P10_m int_, while in the assay with ATP, UTP, and CTP as substrates, the P10 and P13_m_ fraction intensities were calculated as P10_int_/T30_int_ and P13_m int_/P10_int_, respectively. In the single-nucleotide-incorporation assay, the fraction of P10_m_ intensity was calculated as P10_m int_/(P10_m_ + P9)_int_.
