# Supplementary material for: RNA-Dependent RNA Polymerase of the Second Human Pegivirus Exhibits a High-Fidelity Feature
Source: Microbiol Spectr. 2022 Aug 18;10(5):e02729-22. doi: 10.1128/spectrum.02729-22 (PMC9603181; doi:10.1128/spectrum.02729-22)
Supplement: Supplemental file 1 — Fig. S1 to S3. Download spectrum.02729-22-s0001.pdf, PDF file, 0.2 MB [file spectrum.02729-22-s0001.pdf]

Figure S1.

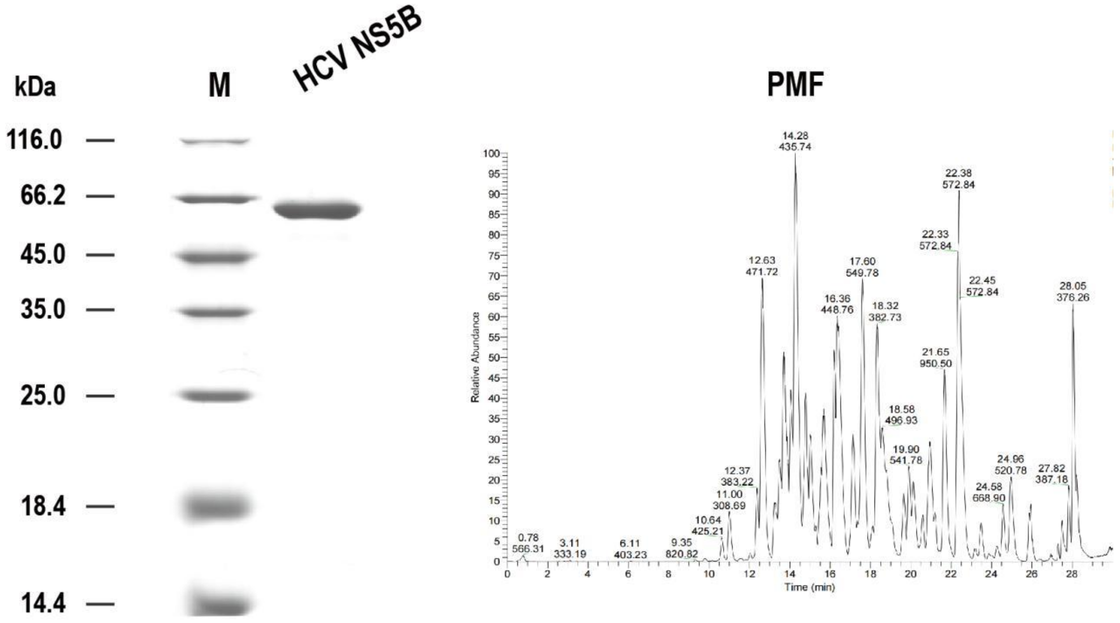

Figure S1. SDS-PAGE and PMF analysis of the purified HCV NS5B. The HCV NS5B was purified sequentially with Ni-NTA chromatography, cation exchange chromatography and gel filtration chromatography. After purification, the protein was confirmed by SDS-PAGE gel electrophoresis and PMF analysis.

Figure S2

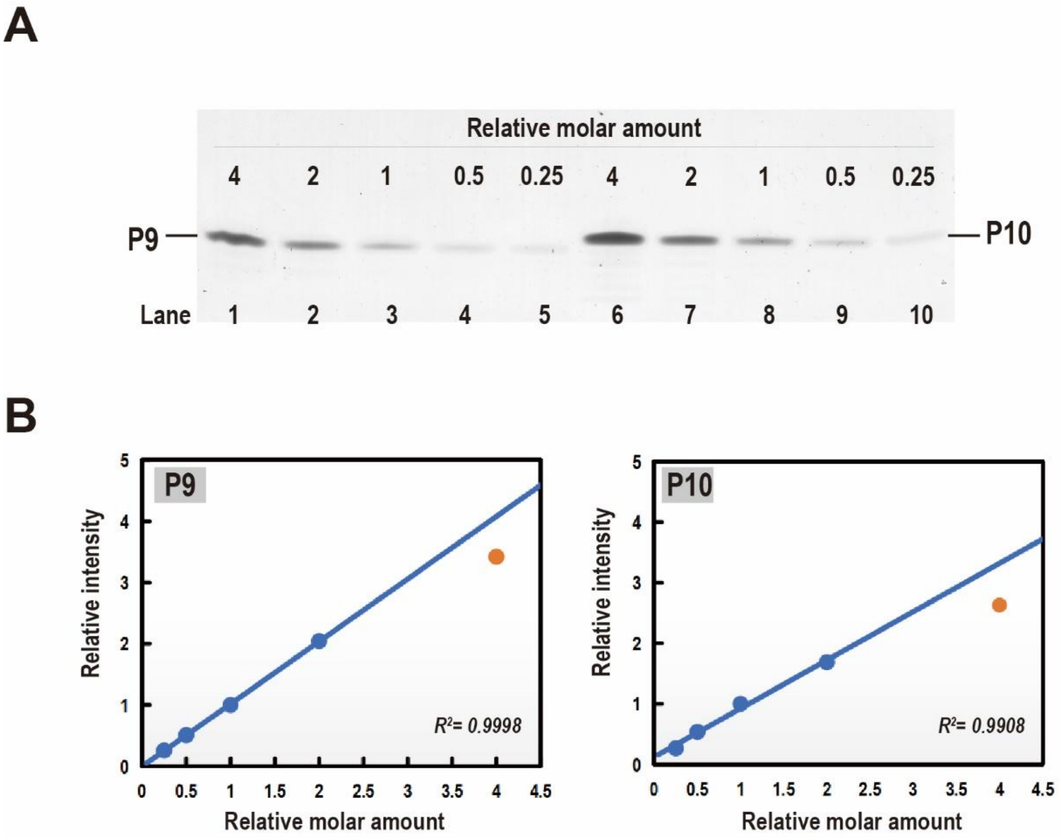

Figure S2. Linear relationship analysis between RNA amount and intensity under stains-all dye. RNA oligos of P9 (GGAUAUAAU) and P10 (GGAUAUAAUC) were chemically synthesized and diluted into a series of concentrations of 3.25, 6.25, 12.5, 25 and 50 μM. The samples were loaded and resolved by 20% polyacrylamide-7 M urea gel electrophoresis and stained with stains-all. A) 20% Urea-PAGE of P9 and P10 at different concentrations. The loading amount of the middle concentration of 12.5 μM was set as 1, and the relative molar amounts of other four concentrations were as indicated. B) Linear relationship analysis of RNA amount and measured intensity. A very good linear relationship between RNA amount and the measure intensity was observed for both P9 and P10 at the concentrations of 3.25 ~ 25 μM ($R^2 > 0.99$), but the intensity of RNA at high concentration of 50 μM become a little deviated. The experiment has been repeated for twice. These results are consistent with our previous study of Wu J in 2015, indicating the stains-all dye is suitable for semi-quantitative analysis.

Figure S3

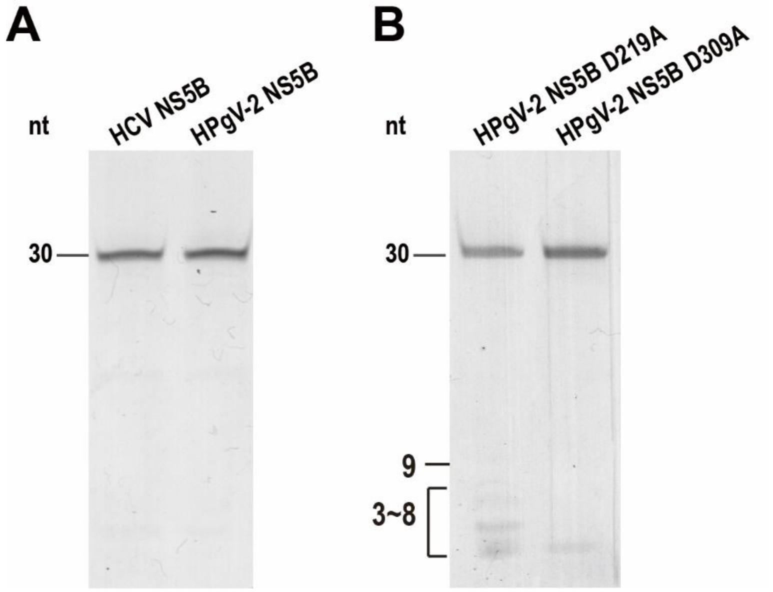

Figure S3. A) The purified proteins were incubated with the RNA template T30 at 30 ºC for 4 hours before applied to the polymerase assays to make sure that there is no RNase contamination and the purified proteins would not induce degradation of the template RNA. B) Catalytic activity of the HPgV-2 NS5B mutants D219A and D309A. D219 and D309 are two critical catalytic aspartates of HPgV-2 polymerase, of which the D219 is responsible for Mn^{2+} conjunction and D319 is located in the active core site (HGDD). Mutations in these two residues results in obvious decrease (D219A) or completely loss (D309A) of catalytic activity. This result (B) can be a negative control for the polymerase assays.
